# Metabolism of Albumin after Continuous Venovenous Hemofiltration in Patients with Systemic Inflammatory Response Syndrome

**DOI:** 10.1155/2015/917674

**Published:** 2015-01-14

**Authors:** Yu Chen, Jianan Ren, Xiaodong Qin, Guanwei Li, Bo Zhou, Guosheng Gu, Zhiwu Hong, JiYe Aa, Jieshou Li

**Affiliations:** ^1^Department of Surgery, Jinling Hospital, Medical School, Nanjing University, 305 East Zhongshan Road, Nanjing, Jiangsu 210002, China; ^2^Minimally Invasive Department of General Surgery, Fushun Central Hospital, Fushun, Liaoning 113006, China; ^3^Lab of Metabolomics, Key Lab of Drug Metabolism and Pharmacokinetics, China Pharmaceutical University, Jiangsu 210002, China

## Abstract

*Background*. The systemic inflammatory response syndrome (SIRS) is characterized by a hypercatabolic state induced by inflammatory mediators. Continuous venovenous hemofiltration (CVVH) stabilizes the internal environment but also aggravates loss of amino acids. The effect of CVVH on protein dynamics is largely unknown. We adopted the stable isotopic tracer technology to investigate how CVVH changed serum albumin metabolism. *Methods*. Twenty SIRS patients were randomized into low- (2000 mL/h) and high- (4000 mL/h) volume CVVH groups according to the rate of replacement fluid. Eight patients with abdominal infection matched for age, sex, and laboratory index served as controls. Consecutive arterial blood samples were drawn during a primed-constant infusion of two stable isotopes to determine the albumin fractional synthesis rate (FSR) and fractional breakdown rate (FBR). *Results*. Before treatment, there was no significant difference of FSR and FBR among 3 groups. After CVVH, the albumin FSR in high- and low-volume groups was 7.75 ± 1.08% and 7.30 ± 0.89%, respectively, both higher than in the control (5.83 ± 0.94%). There was no significant difference in albumin FBR after treatment. *Conclusions*. Protein dynamic indicators could reflect protein synthesis and breakdown state directly and effectively. CVVH increased albumin synthesis, while the breakdown rate remained at a high level independently of the CVVH rate.

## 1. Introduction

The systemic inflammatory response syndrome (SIRS) is an inflammatory state affecting the whole body just characterized by a hypercatabolic state induced by inflammatory mediators [1–6]. Continuous venovenous hemofiltration (CVVH) redistributes the nutritive material in the circulation, stabilizes the internal environment by removing inflammatory mediators and metabolic waste, regulating fluid and electrolyte, and reduces loss of lean body mass induced by the hypercatabolic state [7, 8]. However, CVVH may aggravate the loss of nutritive materials such as amino acids used in protein synthesis, which may exacerbate the hypercatabolic state [9–11]. Therefore, it is imperative to clarify how CVVH affects the protein synthesis and breakdown.

The common methods of observing and assessing protein metabolism including biochemical analysis, Kjeldahl method [12], radioimmunoassay [13], proteomics, and Western blot, fail to dynamically measure protein expression in specific cells or tissues, while the stable isotopic tracer method is able to dynamically assess protein metabolism, by analyzing isotopic enrichment of labeled amino acids and calculating the albumin fractional synthesis rate (FSR) and fractional breakdown rate (FBR) using mathematical model [14–20].

On one hand, CVVH stabilizes the internal environment by removing inflammatory mediators and metabolic waste, which is conducive to protein synthesis; on the other hand, CVVH may aggravate loss of amino acids, which hinders protein synthesis. The combined effect of CVVH on protein metabolism may depend on the relative strength of the two events. This study adopted the stable isotopic tracer technology to carry out isotope infusion trials on SIRS patients treated with CVVH. We investigated the influence of CVVH treatment on albumin FSR and FBR, to explore how CVVH changed serum albumin metabolism, so as to provide some basis for clinical nutrition therapy.

## 2. Materials and Methods

### 2.1. Patients Inclusion

Patients admitted to Jingling Hospital from December 2012 to December 2013 were eligible for inclusion if they were aged 18–70 and met the diagnostic criteria of SIRS [1]: (1) body temperature below 36°C or above 38°C; (2) heart rate faster than 90 beats per minute; (3) tachypnea over 20 breaths per minute, or arterial partial pressure of carbon dioxide below 4.3 kPa (32 mmHg); (4) leukocytes below 4 × 10^9^ cells/L or over 12 × 10^9^ cells/L, or the presence of over 10% immature neutrophils. Patients were not eligible if they had septic shock, chronic renal failure, some main organs failure, active bleeding, heart disease, macroangiopathy, or unstable haemodynamics, or if they were treated with CVVH less than 24 hours or had history of CVVH treatment. CVVH-treated patients were divided into low-volume (2000 mL/h) and high-volume (4000 mL/h) groups according to the random number table. SIRS patients with abdominal infection matched for age, gender, body mass index, and laboratory index served as controls. This study was approved by the Ethics Review Board of Jinling Hospital, Nanjing, China. Written informed consent to participation in the study was obtained from all subjects after the purposes and potential risks of the experimental procedures had been explained in detail.

### 2.2. Experimental Protocol

**Figure 1:**
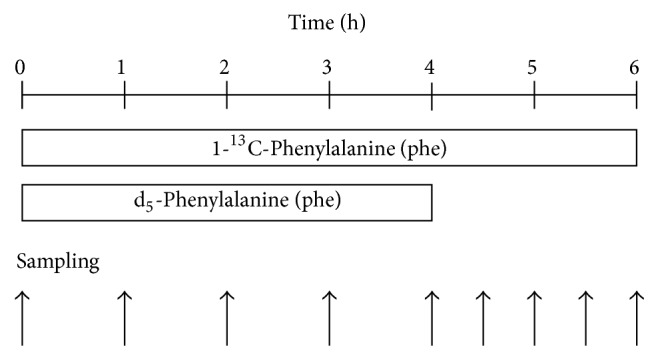
Stable isotope infusion study protocol in control (*n* = 8), low-volume (*n* = 10), and high-volume (*n* = 10) groups. Phe, phenylalanine.

Measurement of albumin synthesis in CVVH treatment group was performed before and 24 h after CVVH treatment. In the control group, it was performed before and after conventional antibiotic and symptomatic treatment. The protocol of the primed-constant infusion of stable isotopes is graphically depicted in Figure 1. A sterile solution of stable isotope-labeled [1-^13^C]Phe and d_5_-Phe was made in 0.45% saline. An intravenous catheter was placed in an antecubital vein for the infusion of stable isotopically labeled amino acid and a second catheter in the radial artery of the contralateral arm for blooding sampling. The hand was kept warm with a heating blanket to relieve discomfort. On starting the infusion study, a priming dose of stable isotopes was given containing [1-^13^C]Phe (6 umol*·*kg^−1^), d_5_-Phe (6 umol*·*kg^−1^) and followed immediately by a constant infusion of tracer [1-^13^C]Phe (6 umol*·*kg^−1^ *·*min^−1^), d_5_-Phe (6 umol*·*kg^−1^ *·*min^−1^). The infusion of [1-^13^C]Phe continued for 6 h, and the infusion of d_5_-Phe for 4 h (Figure 1). Blood samples (3 mL each) were collected hourly for 6 h with two additional samplings at 4.5 and 5.5 h. The samples were drawn into prechilled tubes containing ethylenediaminetetraacetic acid (EDTA) and centrifuged immediately at 4°C; the plasma was stored at −80°C until further analysis.

### 2.3. Analytical Procedures

#### 2.3.1. Precursor Pool Enrichment

According to a previous study [21], for measurement of plasma free phenylalanine enrichment, plasma samples were initially deproteinized by adding 10 microliters of 5-sulphosalicylic acid solution (45%) to 100 microliters of sample. After vortex mixing, samples were centrifuged at 1000 rpm for 10 min at 4°C. The supernatant was acidified to pH3 and loaded to a solid phase extraction (SPE) cartridge. Amino acids trapped on the cartridge were washed out by 2 mL of 3 mol/L ammonia water, and effluents were collected and dried in vacuo.

#### 2.3.2. Albumin Pool Enrichment

The plasma was precipitated with cold 10% TCA and centrifuged. The acid was poured off and cold absolute ethanol added to dissolve the protein pellet. The resultant supernatant containing only albumin was dried under vacuum. To remove trace of free amino acids, the residue was suspended in 0.3 mol/L sodium hydroxide at 37°C for 1 h and reprecipitated in 2 mol/L perchloric acid (HClO_4_). After being washed with 0.2 mol/L HClO_4_, albumin was hydrolyzed with 6 mol/L hydrogen chloride in an evacuated sealed tube at 110°C for 24 h. The hydrolysates were further purified by passing through a 0.22 micron filter and were evaporated to dryness for measurement of isotopic enrichment.

#### 2.3.3. Mass Spectrometry

The purified amino acids from plasma, as well as albumin hydrolysates, were both derivatized with N-methyl-N-trimethylsilyltrifluoroacetamide plus 1% trimethylchlorosilane at 37°C for 30 min. The isotopic enrichment of [1-^13^C]Phe and d_5_-Phe from the plasma free amino acid pool and from albumin was measured by gas chromatography mass spectrometry analysis (model 5973; Hewlett-Packard, Palo Alto, CA) in the electron impact ionization mode. The enrichment was expressed as tracer (labeled amino acids) to tracee (unlabeled amino acids) ratio (TTR). The TTR of [1-^13^C]Phe was expressed by 219/218(*M* + 1/*M* + 0), which is the ratio of mass-to-charge ratio; the TTR of d_5_-Phe was expressed by 197/194(*M* + 5/*M* + 2).

### 2.4. Calculation

As described by Martini et al. [22], plasma albumin FSR was calculated using the formula: FSR = [EB(*t*_2_) − EB(*t*_1_)]/(EF × *t*), where EB(*t*) was the isotopic enrichment of albumin-bound phenylalanine, EF was plasma free phenylalanine enrichment at steady state, *t*_1_ is the time point when d_5_-Phe infusion stopped (4 h), *t*_2_ is the time point when [1-^13^C]Phe infusion stopped (6 h), and *t* is the time interval.

Plasma albumin FBR was determined by calculating the fractional rate of loss of labeled albumin after d_5_-Phe tracer stopped. When d_5_-Phe tracer stopped, the plasma phenylalanine enrichment did not immediately go to zero because it took time for the labeled phenylalanine to be cleared from the blood; therefore labeled albumin continued to be produced during this period. To account for this continued label incorporation, we determined the predicted albumin-bound d_5_-Phe enrichment (assuming that there was no loss of labeled phenylalanine (denoted EB_pred_)) using the formula: EB_pred_ = EB(*t*_1_) + FSR × EF × *t*, where EB(*t*_1_) is the initial albumin-bound phenylalanine enrichment when d_5_-Phe infusion stopped, EF is the enrichment of plasma phenylalanine, and t is the amount of time that has elapsed since the start of albumin-bound phenylalanine measurement. To account for the delay between the time when plasma phenylalanine is taken up to the time when it appears in bound albumin, EF is the free phenylalanine enrichment 1 h before the time of the albumin-bound enrichment.

The actual measured albumin-bound phenylalanine enrichment (denoted EB_act_) will be lower than the above predicted enrichment to the extent that the label is irreversibly lost. Therefore, the FBR can be calculated using the formula: FBR = (EB_pred_ − EB_act_)/[EB(*t*_1_) × *t*]. The numerator of this equation represents the amount of tracer that is irreversibly lost (relative to the amount of tracee). Dividing this value by the initial enrichment [EB(*t*_1_)] gives the percentage of the initial enrichment that is irreversibly lost, and dividing also by the time gives the fractional rate that the tracer is lost. It is assumed that the fractional rate of loss of unlabeled albumin is the same as the labeled albumin.

### 2.5. Statistical Analysis

Data are presented as means ± SD unless otherwise noted. Categorical variables were analyzed by Chi-square test or Fisher exact test, continuous variables by one-way ANOVA, and post hoc LSD test. All statistical analyses were performed with SPSS 19.0 and *P* values < 0.05 were considered statistically significant.

## 3. Results

### 3.1. Subject Characteristics

The subjects' demographics and laboratory index are presented in Table 1. There were 28 SIRS patients, 17 males and 11 females, aged from 31 to 66. Low-volume group (2000 mL/h) consisted of 10 patients with an average age of 46 ± 10 and BMI of 23.07 ± 5.19. High-volume group (4000 mL/h) consisted of 10 patients with an average age of 52 ± 10 and BMI of 22.56 ± 3.22. Control group consisted of 8 patients with an average age of 51 ± 12 and BMI of 23.65 ± 4.37. Demographics and laboratory index showed no statistical difference among 3 groups.

### 3.2. Isotope Data

**Figure 2:**
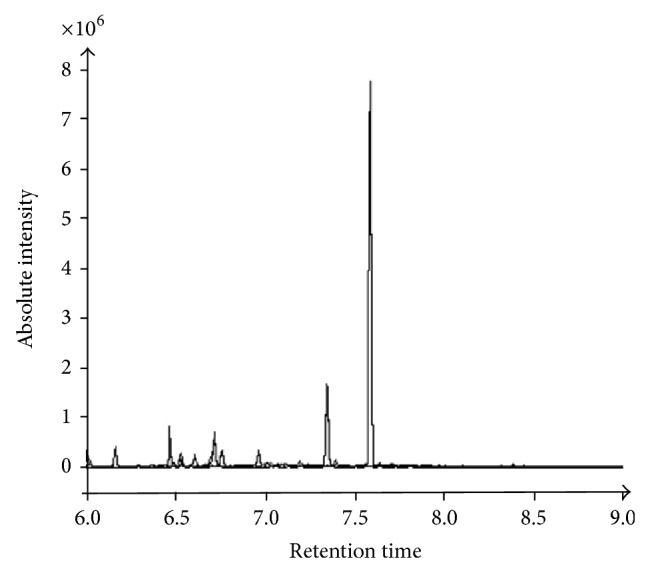
GC/QMS SCAN chromatogram of phenylalanine derivatives. Retention time on the horizontal axis and ionic strength on the vertical one. External ion peak appeared at 7.3 min and phenylalanine derivatives appeared at 7.6 min.

According to the GC/QMS SCAN chromatogram of phenylalanine ethyl esters (Figure 2), external ion peak appeared at 7.3 min of retention time and phenylalanine ethyl esters appeared at 7.6 min, with much more remarkable peak height and area than other substances. Hence, we proceeded to use SIM mode to observe the phenylalanine ethyl esters at 7.6 min of retention time.

**Figure 3:**
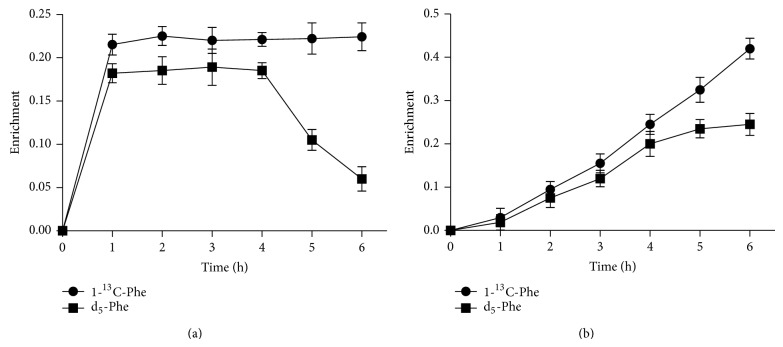
(a) Changes in plasma free Phe enrichments during the infusion of [1-^13^C]Phe (6 h) and d_5_-Phe (4 h); (b) Changes in albumin-bound Phe enrichments during the infusion of [1-^13^C]Phe (6 h) and d_5_-Phe (4 h).

The enrichments of plasma free [1-^13^C]Phe and d_5_-Phe TTR reached plateau values after 1 h of Phe infusion, and the enrichments of plasma free d_5_-Phe TTR slowly decreased after d_5_-Phe infusion stopped between 4 h and 6 h (Figure 3(a)). The enrichment of albumin-bound [1-^13^C]Phe and d_5_-Phe TTR showed a linear increase during the infusion of Phe, and the increase of d_5_-Phe TTR slowed after 4 h (Figure 3(b)). This slowdown indicated the difference between predicted enrichment and actual enrichment that could be used to calculate albumin FSR and FBR.

### 3.3. Albumin Kinetics

**Figure 4:**
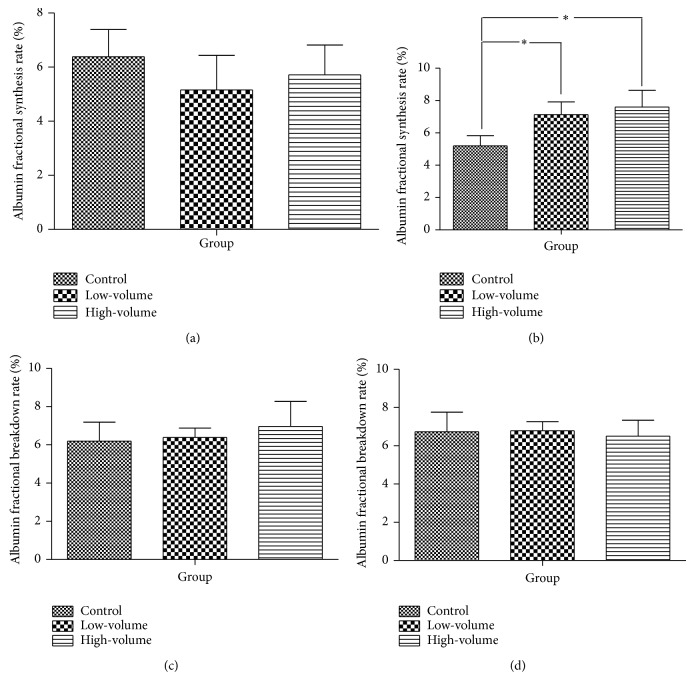
Albumin synthesis rates and breakdown rates in control (*n* = 8), low-volume (*n* = 10), and high-volume (*n* = 10) groups before (a, c) and after (b, d) treatment.

Plasma albumin FSR was calculated from the increase of albumin enrichments between 4 h and 6 h of the infusion. Before treatment, albumin FSRs were 6.38 ± 1.01%/h, 5.16 ± 1.28%/h, and 5.72 ± 1.10%/h, respectively in control, low-volume, and high-volume groups (*F* = 2.528, *P* = 0.100) (Figure 4(a)), showing no significant difference. After treatment, albumin FSRs were 5.20 ± 0.63%/h, 7.13 ± 0.78%/h, and 7.60 ± 1.03%/h, respectively in control, low-volume, and high-volume groups (*F* = 19.357, *P* = 0.000) (Figure 4(b)), with both the high-volume and low-volume groups showing much higher results than the control group (*P* = 0.000). However, there was no significant difference between high- and low-volume groups (*P* = 0.228). CVVH treatment could promote protein synthesis probably by restoring disrupted homeostasis since it reduced inflammatory mediators and metabolic waste. However, volumes of CVVH treatment made no difference.

Plasma albumin FBR was calculated from the changes in albumin-bound Phe enrichment TTR after the cessation of tracer d_5_-Phe infusion. Before treatment, the FBRs were 6.20 ± 0.99%/h, 6.39 ± 0.49%/h, and 6.96 ± 1.31%/h, respectively in control, low-volume, and high-volume groups (*F* = 1.491, *P* = 0.245) (Figure 4(c)). After treatment, the FBRs were 6.74 ± 1.02%/h, 6.79 ± 0.47%/h, and 6.50 ± 0.84%/h, respectively in control, low-volume, and high-volume groups (*F* = 0.357, *P* = 0.703) (Figure 4(d)). No significant difference of albumin FBR was found among 3 groups before and after treatment, which showed that CVVH could not reduce the high protein breakdown rate of SIRS patients.

## 4. Discussion

In this study, we used the technique of mixed infusion of multi-isotopes that allowed simultaneous and independent quantification of albumin synthesis and breakdown in vivo. We found that CVVH treatment promoted albumin synthesis of SIRS patients regardless of the fluid volume. The facilitated albumin synthesis may be attributed to the reduction of inflammatory mediator and metabolic waste via CVVH. However, CVVH treatment failed to reduce the high breakdown rate of albumin in SIRS patients. The improvement of organ function and circulation state after CVVH leads to greater need of amino acids for protein synthesis, whereas several metabolic substrates are diminished during the process of CVVH. Therefore the raw material for protein synthesis is lacking. Under this circumstance, the breakdown rate of albumin in the circulating blood remains at a high level probably to ensure protein synthesis of vital organs. However, this hypothesis needs to be further confirmed.

Study of protein dynamics based on the stable isotopic tracer technology is a branch that straddles proteomics and metabonomics. It connects the two subjects by tracing the synthesis, breakdown, and conversion process of small substrate molecules, which is also the mainline of our study. A few research institutions have reported relevant studies. For example, Martini et al. [22] reported the acute changes in fibrinogen metabolism and coagulation after hemorrhage; Zhang et al. [23, 24] reported muscle protein metabolism of New Zealand rabbit. However, few have ever evaluated synthesis and breakdown rates of plasma albumin simultaneously.

We chose albumin as the observation index for several reasons. Firstly, it has been demonstrated that inflammatory response leads to abnormal metabolism of albumin [25]. Secondly, albumin can be used as index to assess individual nutrition status to some extent. Therefore study of its metabolism can provide some basis for clinical nutrition therapy. Lastly, it is more convenient to extract and purify albumin than any other plasma protein. We tried to evaluate the albumin dynamics in the process of CVVH. However, the clearance function of the filter influenced the precursor pool metabolism, and a pause occurred when the hemofiltration fluid was replaced, thus affecting the application of the mathematical model. Since these interferences could not be eliminated, we turned to studying albumin dynamics 24 h after CVVH.

## 5. Conclusions

Mixed infusion of multi-isotopes for simultaneous measurement of plasma albumin synthesis and breakdown rate is an effective and feasible method of albumin dynamic assessment, which is worthy of being applied more extensively. CVVH removes metabolic wastes, regulates body fluids, maintains acid-base balance, and improves the immune homeostasis, thus increasing albumin synthesis rate, while albumin breakdown rate remains at a high level probably to ensure protein synthesis of vital organs. However, further studies are warranted to confirm this hypothesis.

## Figures and Tables

**Table 1:** Demographics and laboratory index of patients.

	Control (*n* = 8)	Low-volume (*n* = 10)	High-volume (*n* = 10)	Statistics	*P* value
Age	51 ± 12	46 ± 10	52 ± 10	*F* = 0.79	0.466
Gender (male)^*^	62.50%	60.00%	60.00%	*χ* ^2^ = 0.02	0.993
BMI	23.65 ± 4.37	23.07 ± 5.19	22.56 ± 3.22	*F* = 0.14	0.869
APACHE II	11.88 ± 3.18	13.60 ± 3.63	14.70 ± 3.34	*F* = 1.54	0.234
WBC (×10^9^)	11.95 ± 4.99	17.19 ± 9.09	12.03 ± 7.48	*F* = 1.53	0.237
PLT (×10^12^)	98.50 ± 34.40	94.60 ± 35.60	92.80 ± 38.78	*F* = 1.30	0.291
TP (g/L)	54.00 ± 15.86	56.40 ± 13.36	54.20 ± 13.98	*F* = 0.08	0.923
ALB (g/L)	38.00 ± 7.40	36.10 ± 3.21	36.60 ± 5.04	*F* = 0.30	0.745

BMI: body mass index. APACHE II: acute physiology and chronic health evaluation score II. WBC: white blood cell. PLT: platelet. TP: total protein. ALB: albumin. ^*^Gender stands for the percentage of male patients.
